# Physically constrained voxel‐based penalty adaptation for ultra‐fast IMRT planning

**DOI:** 10.1120/jacmp.v17i4.6117

**Published:** 2016-07-08

**Authors:** Niklas Wahl, Mark Bangert, Cornelis P. Kamerling, Peter Ziegenhein, Gijsbert H. Bol, Bas W. Raaymakers, Uwe Oelfke

**Affiliations:** ^1^ Department of Medical Physics in Radiation Oncology German Cancer Research Center ‐ DKFZ Heidelberg Germany; ^2^ Joint Department of Physics The Institute of Cancer Research and The Royal Marsden HS Foundation Trust London UK; ^3^ Department of Radiotherapy University Medical Center Utrecht The Netherlands

**Keywords:** dose optimization, importance factors, IMRT, inverse planning, adaptive radiation therapy

## Abstract

Conventional treatment planning in intensity‐modulated radiation therapy (IMRT) is a trial‐and‐error process that usually involves tedious tweaking of optimization parameters. Here, we present an algorithm that automates part of this process, in particular the adaptation of voxel‐based penalties within normal tissue. Thereby, the proposed algorithm explicitly considers *a priori* known physical limitations of photon irradiation. The efficacy of the developed algorithm is assessed during treatment planning studies comprising 16 prostate and 5 head and neck cases. We study the eradication of hot spots in the normal tissue, effects on target coverage and target conformity, as well as selected dose volume points for organs at risk. The potential of the proposed method to generate class solutions for the two indications is investigated. Run‐times of the algorithms are reported. Physically constrained voxel‐based penalty adaptation is an adequate means to automatically detect and eradicate hot‐spots during IMRT planning while maintaining target coverage and conformity. Negative effects on organs at risk are comparably small and restricted to lower doses. Using physically constrained voxel‐based penalty adaptation, it was possible to improve the generation of class solutions for both indications. Considering the reported run‐times of less than 20 s, physically constrained voxel‐based penalty adaptation has the potential to reduce the clinical workload during planning and automated treatment plan generation in the long run, facilitating adaptive radiation treatments.

PACS number(s): 87.55.de

## I. INTRODUCTION

The goal of every radiation treatment is the homogeneous irradiation of cancerous tissue at the best possible rate while sparing healthy tissue. In intensity‐modulated radiation therapy (IMRT), this can be achieved with modulated fields applied from several beam directions.

The IMRT treatment planning process relies on solving an inverse problem that finds adequate radiation fluences according to a predefined dose prescription to cancerous target volumes (e.g., Bortfeld^(1)^ and references therein). The ideal solution — an irradiation of the target without any dose in healthy tissue — is physically impossible to reach due to the nature of radiation transport to the target. Thus, it can merely be approximated by optimizing the beam fluences with cost functions defining a trade‐off between coverage of the target and exposure of critical structures.

The clinical quality of an optimized plan strongly depends on the formulation of the cost function and on the selection of the respective optimization parameters. The arguably most common formulation of the cost function F has been the penalized sum of piecewise squared dose deviations to the prescribed dose of the presegmented volumes of interest (VOIs),^(2^) given by

(1) $$F=\sum_{V}\frac{1}{n_{V}}\left[P_{V}^{\max}\cdot \sum_{i\in V}f_{i}^{\max}+p_{V}^{\min}\cdot \sum_{i\in V}f_{i}^{\min}\right]$$

with

(2) $$f_{i}^{\max}(d_{i})={\left\{d_{i}-d_{V}^{\max}\right\}}_{+}^{2}\;\text{and}\;f_{i}^{\min}(d_{i})={\left\{d_{V}^{\min}-d_{i}\right\}}_{+}^{2}$$

In the given formulation, the prescribed dose to the respective VOI, V, is replaced by the reference dose constraints $d^{\text{max}}$ and $d^{\text{min}}$ for which the positivity operator {·}+ ensures that only the appropriate deviations to the dose $d_{i}$ in voxel i contribute to F. The notation i∈V restricts the summation to voxels i that belong to VOI V. The squared deviations from Eq. (2) are weighted by corresponding penalty weighting factors $p^{\text{max}}$ and $p^{\text{min}}$. For each VOI, the sum of penalized squared deviations is normalized by the corresponding number of voxels $n_{V}$.

Minimization of a quadratic objective function results in a mathematically optimal result. However, the notion of quadratic deviation in itself and the inability to shape local dose distributions does not necessarily yield a clinically optimal dose distribution since the reference doses and penalty weighting factors are abstract and VOI‐based constructs without direct clinical significance. ${p_{V}}^{\text{min}/\text{max}}$ and ${d_{V}}^{\text{min}/\text{max}}$ are thus indirect tools to shape a dose distribution according to the planner's wishes. Manually finding a suitable set of dose constraints and penalty weighting factors is a tedious and time‐consuming process. The automation of this process is often tackled with computationally expensive multicriteria optimization.^3^, ^4^, ^5^ In clinical practice, however, an automated and fast planning process is desirable to reduce the workload of the treatment personnel and enable novel adaptive treatment strategies.

Ziegenhein et al.^(6)^ developed an ultra‐fast optimization software module that is capable of reducing the optimization time for a single treatment plan from several minutes to a few seconds. In this paper, we take advantage of this ultra‐fast optimization by incorporating a voxel‐based penalty adaptation algorithm in our planning software to enhance and automate the decision making process of choosing adequate penalties ${p_{V}}^{\text{min}/\text{max}}$.

Several algorithms that adapt voxel‐based parameters like penalty and reference doses or directly manipulate the associated beamlet weights have been proposed since 2000.^(5,7–17^) Recently Zarepisheh et al.^(18)^ explained the mathematical foundation of these algorithms. For the purpose of this paper, we identify two different classes of adaptation strategies. Algorithms of the first class^5^, ^9^, ^10^, ^13^ focus on the decision‐making problem and thus aim for an optimization of the importance factors, subject to additional objectives than the actual objective function used during inverse planning. The second class of adaptation algorithms^7^, ^8^, ^11^, ^12^ aims for a fast generation of an acceptable plan by adapting the penalties of a previous nonacceptable plan heuristically. This possibly enables class solutions, which is a set of optimization parameters suitable for comparable cases of the same indication, and online adaptive treatment planning, or provides the planner with more interactive possibilities and freedom.

In this study we present ΦWA, a novel voxel‐based penalty adaptation strategy of the second class. The acronym stands for ‘Physical Weight Assignment’ and is an extension of the previously proposed ‘Dynamic Importance Weight Assignment’ (DIWA).^(7^) Like DIWA, ΦWA uses a heuristic approach to calculate voxel‐based penalty distributions based on the resulting dose distribution of a conventionally optimized treatment plan. ΦWA features a reproducible adaptation strategy needing a few iterations. It extends DIWA to explicitly account for the physical characteristics of photon beams within the patient. We evaluate the adaptation strategy with a planning study on 16 prostate and 5 head and neck cancer patients.

The remainder of this paper is organized as follows: Section II introduces the mathematical and physical concepts used by ΦWA, the computational implementation and the layout of the treatment planning study utilized to evaluate the efficacy of the developed methodology. The results are presented in Section III. Sections IV and V discuss and conclude this paper.

## II. MATERIALS AND METHODS

### A. Planning and optimization procedure

#### A.1 Initial optimization

The planning process of ΦWA begins with a conventional optimization of the objective function F (Eq. (1)) with respect to the beam fluences. These are represented by beamlets j with a corresponding weight $w_{j}$, which generate the dose $d_{i}$ through a precalculated dose influence matrix $D_{\text{ij}}d_{i}=\sum_{j}^{j}D_{\text{ij}}w_{j}$. The solution w* to the optimization problem is then given by

(3) $$w^{*}=\underset{w\in {\mathbb{R}}_{+}^{n_{b}}}{\arg\;\min}\;F(w)$$

where $n_{b}$ is the number of beamlets and ${\mathbb{R}}_{+}^{n_{b}}$ denotes the real positive orthant.

#### A.2 Modification of the objective function

The optimization is followed by the calculation of the new voxel‐based penalty scale. The distribution of this voxel‐based penalty scale $\phi_{i}$ will be used for a reoptimization which generates an adapted plan based on a modified objective function

(4) $$\tilde{F}=\sum_{V}\frac{1}{n_{V}}\left[P_{V}^{\max}\cdot \sum_{i\in V}\varphi_{i}f_{i}^{\max}+p_{V}^{\min}\cdot \sum_{i\in V}\varphi_{i}f_{i}^{\min}\right]$$

In general, the process of adaptation and reoptimization with $\tilde{F}$ can be repeated. However, few repetitions are desired to guarantee short run‐times.

#### A.3 Heuristics for penalty adaptation

The underlying idea of ΦWA is to increase the penalty within the healthy tissue by the penalty scale $\phi_{i}^{O}$ such that the local objective function $F_{i}$($d_{i}$) at a voxel i with dose $d_{i}$ of the last optimization matches its hypothetical value for a desired dose threshold $d_{T}$. We label this objective‐based strategy by

(5) $$\varphi_{i}^{O}(d_{i})={\left[\frac{f_{i}^{\min/\max}(d_{i})}{f_{i}^{\min/\max}(d_{T})}\right]}^{\alpha }$$

where α is an additional scaling power. Hence, our method comprises two independent parameters $d_{T}$ and α to define the threshold and the relative strength of the applied scaling. By default, the parameter α equals one.

With the new calculated penalty scale distribution $\phi_{i}^{O}$, high‐dose areas within the healthy tissue of the previous optimization get a locally higher penalty factor used for the reoptimization of $\tilde{F}$. $d_{T}$ defines the threshold above which dose regions are considered as undesirably overdosed.

This elevation of penalties does, however, interfere with the target irradiation by decreasing coverage and homogeneity, which can be seen in the dose‐volume histograms (DVHs) of previous publications^8^, ^11^ and in the Results section below. The reason for this lies in physical properties like penumbra, aperture size and beam set‐up, which do not allow for infinitely steep dose gradients on the one hand, and an exact geometrical match of the fluence projection on the target shape on the other.

In medium and high dose regions the falloff of a photon beam in the lateral direction δ (i.e., the beam penumbra $d_{P}$) can be approximated reasonably well with an error function:^(19,20^)

(6) $$d_{P}(\delta )=\frac{1}{2}\cdot d^{pres}\cdot erfc\left(\frac{\delta }{\sqrt{2}\sigma_{P}}-1.16309\right)$$

where $d^{\text{pres}}$ represents the prescribed dose to the target, and $\sigma_{P}$ is the penumbra width. The subtrahend 1.16309 results from a coverage condition $0.95d^{\text{pres}}=d_{P}(\delta =0)$, meaning that the target should at least receive 95% of its prescribed dose.

Please note that $\sigma_{P}$ depends on the depth and field size. For our study, we considered a constant $\sigma_{P}=3.2\;\text{mm}$ as suggested by van Herk et al.,^(19)^ which is also consistent with measurements.^(21^)

**Figure 1:**
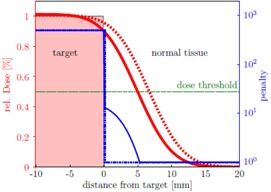
Beam penumbra (Eq. (6)) at the target border (dashed red) using constant and VOI‐based penalties of ${p_{\text{NT}}}^{\text{max}}=1$ in the normal tissue and ${p_{T}}^{\text{min}}=500$ (dotted blue) for the target and reoptimized beam penumbra (solid red) using adapted penalties (solid blue) according to Eq. (5). The dose threshold is shown in solid green. The increased penalty in the penumbra area pushed the penumbra profile towards the target and thus reduces coverage.

Figure 1 illustrates the issue of compromised target coverage by solving the inverse planning problem for an error function beam penumbra in a one‐dimensional case. Apparently, naive scaling of the penalties close to the target volume yields an incentive for the optimizer to reduce the dose outside of the target volume by compromising coverage inside. We conclude that there is a penumbra region where an additional adaptation of the penalties must be prohibited by the algorithm, which we represent by an allowed penumbra distance δP from the target by inverting Eq. (6):

(7) $$\delta_{P}(d)=\sqrt{2}\sigma_{P}\left[\cdot erfc^{-1}\left(\frac{2d}{d^{pres}}\right)+1.16309\right]$$

Furthermore, due to the finite beamlet size and the finite number of beams, the penumbra of a beam does not always “fit” to the geometry at the target border and is in general superimposed by the projected photon depth doses and penumbras of the remaining beams. To give the optimizer enough freedom within these areas, we incorporated an additional region Δδ around the prohibited penumbra region in which the adaptation is gradually released to full strength.

This suppression of the penalty adaptation is realized by extending Eq. (5) with a subtrahend containing a term $S_{i}(d_{i},\delta_{i})$ decreasing with the distance $\delta_{i}$ of voxel i to the target:

(8) $$\varphi_{i}^{S}(d_{i},\delta_{i})=\varphi_{i}^{O}(d_{i})-\left[\varphi_{i}^{O}(d_{i})-1\right]S_{i}(d_{i},\delta_{i})$$

With the condition $0\leq S_{i}\leq 1$, the new subtrahend decreases the penalty scale to 1 (meaning no scaling) as we approach full suppression at small distances ($S_{i}=1$), and has no effect if we have no suppression at large distances ($S_{i}=0$).

We propose a suppression factor that decreases quadratically with distance $\delta_{i}$ where we can quantify the extent of the suppression region by a maximal distance Δδ:

(9) $$S_{i}^{'}(d_{i},\delta_{i})=\{\begin{array}{ll} 1, & \delta_{i}\leq \delta_{P}\\ 1-{\left(\frac{\delta_{i}-\delta_{P}(d_{i})}{\Delta \delta }\right)}^{2}, & \delta_{P}<\delta_{i}<\Delta \delta +\delta_{P}\\ 0, & \delta_{i}\geq \Delta \delta +\delta_{P} \end{array}$$

$S^{\prime }$ is, however, only applicable for $d_{i}<d^{\text{pres}}$, since otherwise evaluation of Eq. (7) is not possible due to the restricted domain of the inverted complementary error function. Since doses $d_{i}\geq d^{\text{pres}}$ within the healthy tissue appear infrequently, we chose to use an empirically determined correction factor for this domain that continues the course of the error function qualitatively and artificially shortens the allowed region Δδ. For doses $0.95d^{\text{pres}}<d_{i}<d^{\text{pres}}$, where $\delta_{P}(d_{i})$ would be negative, we set $\delta_{P}=0$. The full suppression factor $S_{i}$($d_{i}$,$\delta_{i}$) then reads

(10) $$S_{i}(d_{i},\delta_{i})=\{\begin{array}{ll} S^{\prime }(d_{i},\delta_{i}), & d_{i}<d^{pres}\\ 1-{\left(\frac{\delta_{i}}{\Delta \delta }\right)}^{2}\exp\left[5\left(\frac{d_{i}}{d^{pres}}-1\right)\right], & d_{i}\geq d^{pres} \end{array}$$

For high doses $D_{i}$ where Eq. (10) would yield $S_{i}\;<0$, we apply the above introduced condition that $0\leq S_{i}\leq 1$ and consequently set $S_{i}=0$.

**Figure 2:**
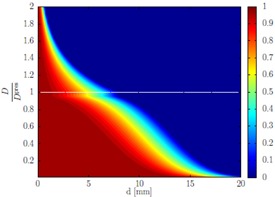
Visualization of the suppression factor S depending on dose relative to the prescribed target dose and distance from target. S=1 means a full suppression of the penalty scaling and thus φ=1. For S=0, the penalty is adapted unimpededly according to Eq. (5). The white line represents the prescribed dose.

The final S(d,δ) and its dependence on dose and distance is visualized in Fig. 2. This physical suppression with $S_{i}$ is the central part of ΦWA.

### B. Implementation

ΦWA has been developed for the research treatment planning software module μKonRad(^6^) and can additionally be accessed through the research treatment planning system DynaPlan^(22)^ via a graphical user interface (GUI).

#### B.1 Optimization module

μKonRad utilizes a highly speed‐optimized L‐BFGS implementation.^(6,23^) The incorporation of a penalty scale $\phi_{i}$ into μKonRad implies only one additional floating point operation within the objective function evaluation during the optimization. Consequently, our algorithm does not yield a substantial increase of the run‐time of the optimization module. Consumption of memory however, is increased due to the added penalty scale cube and necessary calculation of additional distance transforms.

To calculate the distance to the target needed for Eqs. (9) and (10), a 3D extension of the Chamfer distance algorithm (CDA)^24^, ^25^ was implemented. It is applied to the target borders with a pseudo‐Euclidean distance transform that assigns the distances <3,4,5> to the adjacent voxels (nearest to most far), which Borgefors(^25^) identified as the best integer approximation compared to the true Euclidean distance.

Since the physically allowed dose levels depend on the prescribed dose to the target, we group the targets by their prescribed dose. To save memory, we do not calculate the distance transform for each VOI, but only for the respective group of targets which are needed for the current penalty adaptation. The algorithm then chooses the adequate $\delta_{P}$ to use in Eqs. (9) and (10) by selecting the smallest difference $\delta_{i}‐\delta_{P}$ out of all prescription groups.

#### B.2 Graphical user interface

Kamerling and colleagues^(22)^ developed a new interactive treatment planning software which also serves as front‐end for μKonRad with graphical user interface. This enables evaluation and manipulation of the parameters like α, $d_{T}$, and Δδ not only before a planning process, but also in‐between single optimization and penalty adaptation runs. It is furthermore possible to access the three‐dimensional penalty scale cube, providing the possibility to interactively manipulate $\phi_{i}$ (see Section IV).

### C. Planning study

A planning study was performed with 16 prostate cancer patients and 5 more complex head and neck cases. The base data have been provided by UMC Utrecht, and the dose influence matrices $D_{\text{ij}}$ have been calculated with a magnetic field of 1.5 T for adaptive planning with an MR‐LINAC.^(26)^ In the prostate (head and neck) cases, setups of seven coplanar 6 MV beams at 0°, 50°, 100° (80°), 155°, 200° (205°), 260° (280°), and 310° have been used, with bixel sizes of 5 mm. More details on the patient data can be found in Appendix A.

In this study we only applied ΦWA to the body contour. Local penalty adaptation within organs at risk (OARs) is not considered. The reason for this decision was, on the one hand, to measure independently how OARs and targets are affected by changes of the local penalties within the body contour. On the other hand, the adaptation parameters can be transferred more easily from one treatment site to another, since OAR evaluation criteria may vary widely.

For the prostate patients, a detailed parameter study has been performed to analyze the behavior of plan quality depending on the scaling power α, the desired dose threshold $d_{T}$, and the allowed distance Δδ for the respective scaling strategies. The plan quality was evaluated through common quality indicators (QI), including the conformity index (CI)^(27^) and target coverage (CO)

(11) $$CI=\frac{V_{T,95}}{V_{T}}\times \frac{V_{95}}{V_{T}}=CO\times \frac{V_{95}}{V_{T}}$$

where $V_{T}$ is the target volume, $V_{95}$ the overall volume that receives at least 95% of the prescribed target dose, and $V_{T,95}$ is the corresponding subvolume within the target. Furthermore, dose‐volume indicators as proposed by QUANTEC,^(28^) as well as DVHs and the visual inspection of the dose distribution, are evaluated.

Additionally, the adaptation procedures were applied to the prostate and head and neck cases with a predefined class solution optimization constraint set ${p_{V}}^{\text{min}/\text{max}}$, ${d_{V}}^{\text{min}/\text{max}}$ which was the same for all cases of a treatment site, respectively. In this way, we evaluated the capability of the strategies to adapt plans without a plan‐specific, fine‐tuned constraint set.

## III. RESULTS

### A. Parameter study

#### ***A.1 Reoptimization with*** $\varphi^{O}$

**Figure 3:**
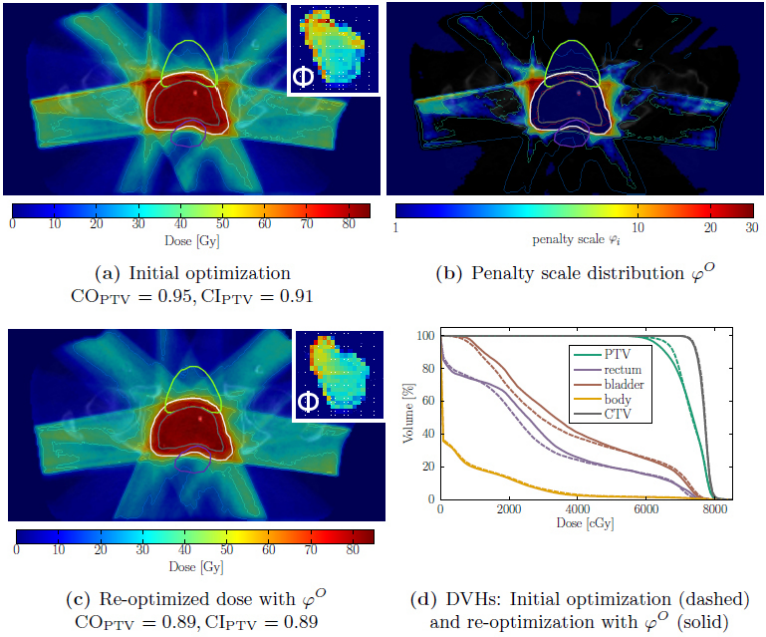
Elimination of high‐dose regions with “naive” voxel‐based penalty adaptation. The initial dose distribution (a) shows several high‐dose regions outside the target. The unconstrained local penalty adaptation routine calculates a high penalty scale for the high‐dose regions, also particularly within the dose fall‐off near the target as the $\varphi^{O}$‐distribution (b) demonstrates. In the reoptimized plan (c), the dose in the former high‐dose regions decreased, but also the coverage, illustrated by the $0.95{d_{\text{PTV}}}^{\text{pres}}$ isodose line, decreased significantly. The shown isodose lines are, from low to high dose: 20 Gy, 35 Gy, 45 Gy, 60 Gy, $0.95{d_{\text{PTV}}}^{\text{pres}}$, $0.95{d_{\text{CTV}}}^{\text{pres}}$, $1.05{d_{\text{PTV}}}^{\text{pres}}$. DVHs for the initial and reoptimized plan are shown in (d). (a) and (c) also contain exemplary fluence maps of one beam (260°) in arbitrary units to visualize changes in the fields' intensity‐modulation.

We illustrate the dose‐dependent heuristic penalty adaptation $\varphi^{O}$ with an example of a dose distribution in Fig. 3. It was efficiently cleared of hot‐spots in the normal tissue, but the penalty adaptation algorithm also selects a very high $\phi_{i}$ for voxels in the immediate target neighborhood, thus reducing target coverage CO from 95% to 89% and conformity CI from 0.91 to 0.89. Additionally, in the adapted plan, smoother fluence maps can be observed.

The loss of coverage observed in Fig. 1 and in an example case in Fig. 3 is substantiated statistically by the parameter study. For the preparation we planned every patient with an individual constraint set (${p_{V}}^{\text{min}/\text{max}}$, ${d_{V}}^{\text{min}/\text{max}}$) to provide a comparable starting situation for all cases. The primary goal of the parameter study was to determine negative effects on the plan quality and to learn which parameter combinations are feasible to minimize these effects.

**Figure 4:**
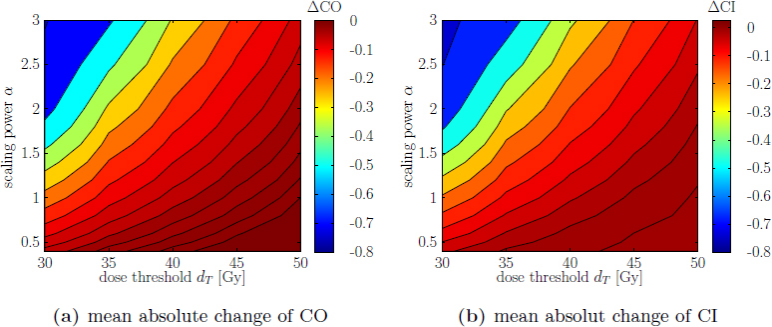
Dependence of PTV CO and CI index changes on $d_{T}$ and α. The plots show the mean absolute change of CO (a) and CI (b) between the reoptimization and the first optimization.

Figure 4 shows the mean absolute change of coverage (CO) and the conformity index (CI) for penalty adaptation with $\phi_{i}^{O}$ (Eq. (5)).

The plots show that certain sets of combinations produce comparable effects on the respective quality indicator and the existence of an isocurve beyond which the indicators start to drop rapidly. Also we can derive that a decrease in conformity can mainly be attributed to the loss of target coverage, as explained in the Materials & Methods section A.3 and illustrated in Fig. 3.

As the DVH in Fig. 3 shows, low‐dose regions within the OARs are slightly increased, while the exposure to high doses is decreased by the penalty adaptation. We could also observe this behavior within the parameter study.

#### A.2 Physical adaptation

**Figure 5:**
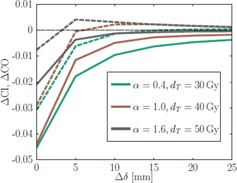
Change of coverage (solid line) and conformity (dashed line) averaged over all patients between the initial and reoptimized plan, depending on the extent of Δδ for three suggestive parameter set.

The dependence of ΔCI and ΔCO on the allowed distance Δδ is shown in Fig. 5 for different parameter settings. A stable behavior of ΔCI and ΔCO can be observed for Δδ≥1 cm. We also want to point out the increase in conformity for some regions.

**Figure 6:**
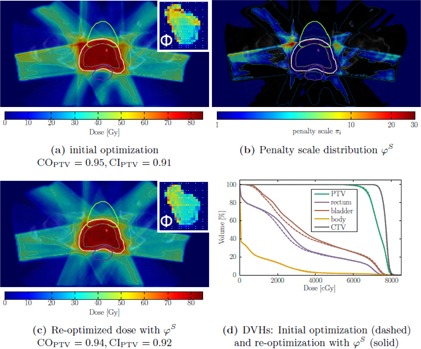
Elimination of high‐dose regions with ΦWA ($\phi^{S}$). The same initial dose (a) as in Fig. 3 is used. Adaptation with $\phi^{S}$ (b) spares the target neighborhood. In this case, the reoptimized plan (c) shows, besides the decreased dose in the respective regions, an almost preserved coverage, also illustrated by the $0.95{d_{\text{PTV}}}^{\text{pres}}$ isodose line. The shown isodose lines are the same as in Fig. 3. The respective DVHs are shown in (d). Again, the fluence maps of the beam at 260° are shown in arbitrary units in (a) and (c).

Figure 6 shows the dose before and after application of $\varphi^{S}$. ΦWA does actually decrease the overall impact on plan quality compared to adaptation with $\varphi^{O}$, since our physical constraining only suppresses the naive adaptation in the respective regions, while still efficiently eliminating undesired high‐dose regions. Furthermore, the improved smoothness of the fluence maps due to adaptation is at least preserved or even increased. Choosing a higher Δδ does decrease the impact of ΦWA further, but a too large tolerance region Δδ will lead to almost no effect when adapting plans with hot‐spots near the target. Thus, Δδ should be chosen to be only large enough to allow the necessary dose falloff near the target. From the stability analysis of the QIs in the parameter study, we suggest a value of around Δδ approximately equal to 10 mm to 15 mm.

### B. Class solutions

#### B.1 Prostate cases

**Figure 7:**
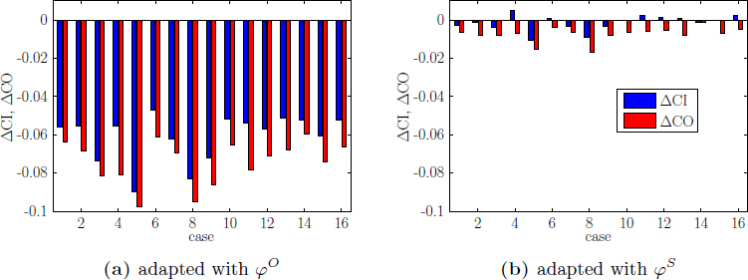
Change of CO and CI for every prostate case for adaptation with $\varphi^{O}$ (a) and $\phi^{S}$ (b).

Based on the parameter study, we decided that for the prostate cases a parameter set of α=1.0, $d_{T}=35\;\text{Gy}$, and Δδ=12 mm provides a reoptimized plan with small enough negative effects on the evaluated quality indicators. This combination of α and $d_{T}$ is chosen since it lies in a region in Fig. 4 that indicates impact on quality indicators while still providing sufficient stability. While we applied individual penalties ${p_{V}}^{\text{min}/\text{max}}$ and reference doses ${d_{V}}^{\text{min}/\text{max}}$ for the parameter study in section A above, we now evaluate the potential of ΦWA to generate class solutions for different indications. Therefore we apply the same set of VOI‐dependent parameters, as shown in Appendix B (Table B.1.), for every patient case. Figure 7 shows the changes in CI and CO for all prostate cases while comparing $\varphi^{O}$ with $\phi^{S}$.

We can again observe that ΦWA efficiently reduces the impact on coverage and subsequently conformity. Since ΔCI>ΔCO for all cases (and sometimes even ΔCI>0), we can derive a decrease of high‐dose regions outside the PTV, indicating successful eliminations of hot‐spots analogous to the dose distributions shown in Figs. 3 and 6.

#### B.2 Head and neck cases

For the five head and neck cancer patients, we again used an empirically determined class‐solution constraint set for the initial optimization (see Appendix B, Table B.2.). The head and neck cases represent more complex planning scenarios. While the planning problem for the prostate case is mainly restricted to rectum, bladder, and target volumes, the anatomy in the head and neck region involves more clinical relevant structures. Multiple target volumes with different prescriptions additionally complicate the situation and all of the initial plans showed undesired hot spots within the healthy tissue. For adaptation with $\phi^{S}$, we again use α=1.0, $d_{T}=35\;\text{Gy}$, Δδ=12 mm to undermine the aforementioned transferability of the method in between treatment sites.

**Figure 8:**
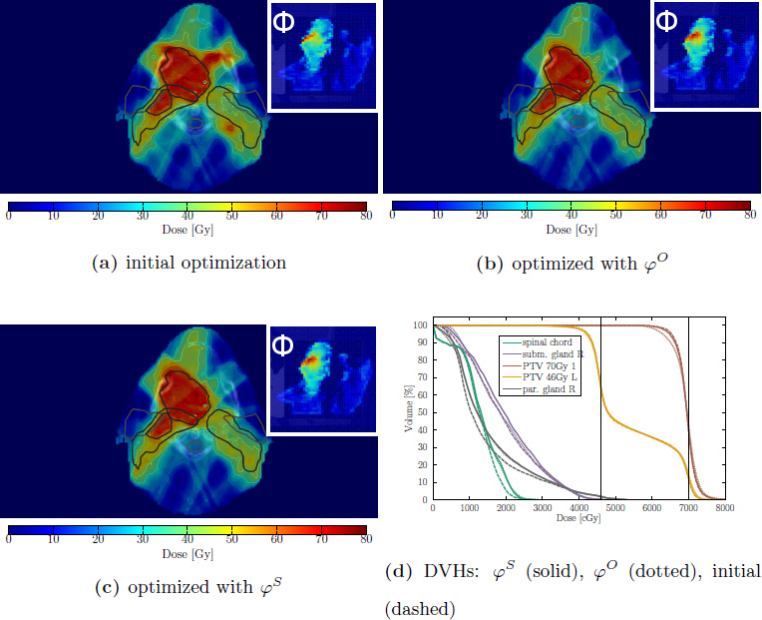
Case 1 optimized with the class solution constraint set (a). Reoptimizations were performed with physical adaptation $\phi^{S}$ (c) and without ($\varphi^{O}$) (b). The DVH in (d) shows the dose volume of two selected targets and the spared parotid and submandibular gland as well as of the spinal cord. The dose distributions again contain the exemplary fluence maps of the beam at 280° in arbitrary units.

The head and neck cases differ in the quantity of irradiated target volumes and their prescribed dose; thus a quantification and interpretation of target conformity through the CI is more difficult, since multiple target structures contribute to the high‐dose tissue and are thus influencing the CI of a single target. Additionally, several target volumes are delineated. Thus, we only present the results by example dose distributions and the corresponding DVHs of the first case in Fig. 8. Complete QI changes can be found in Appendix B, Table B.3.

#### B.3 Convergence characteristics and run‐times

Table 1 shows the progression of the mean squared deviation of the penalty scale distributions of successive adaptation and reoptimization procedures.

Within two runs, the mean squared deviation decreases by two orders of magnitude indicating stability of the scaling distribution after run two. With the run‐times presented in Table 2, we are able to produce an adapted plan within a few seconds.

**Table 1:** Mean squared deviation of the calculated penalty scale distributions after successive adaptation runs to the respective previous run for a prostate and a head and neck case. Run 1 is compared to the distribution initialized with $\phi_{i}=1$ for all voxels

*Case*	*Run 1*	*Run 2*	*Run 3*	*Run 4*	*Run 5*	
p 4	4.942	0.228	0.055	0.033	0.022	$\times 10^{‐2}$
hn 1	3.789	0.128	0.035	0.025	0.017	$\times 10^{‐2}$

**Table 2:** Run‐times in seconds of the ΦWA planning sequence with one reoptimization for a prostate and a head and neck case averaged over 20 respective optimizations with standard deviation, executed on a Windows 7 machine with 32GB of RAM and an Intel Core i7–2600 (3.4 GHz). We evaluated the individual durations of the initial optimization (init. w/φ), the calculation of the penalty scale distribution (φ calc.), the on‐the‐fly calculation of the distance transform (metric calc.), and the reoptimization with φ (reopt). The last column is the sum of these and thus represents the total run‐time of one plan calculation and adaptation. To monitor the performance loss due to the implementation of the additional penalty scale cube, the initial optimization was additionally performed without the ΦWA module enabled (init. w/o φ). The run‐times can be related to the size of the base dataset via Appendix A Table A.1

*Case*	*Init. w/o* φ	*Init. w/*φ	φ *Calc*.	*Metric Calc*.	*Reopt*.	*Total*
p 4	3.85±0.91	3.89±0.91	0.155±0.019	0.371±0.091	2.93±0.69	7.35±1.14
hn 1	7.91±1.82	8.26±1.91	0.196±0.017	1.64±0.02	8.47±1.94	18.6±3.3

## IV. DISCUSSION

The previous sections documented the implementation and evaluation of a physically constrained voxel‐based weight adaptation algorithm ΦWA. The algorithm works stepwise by reoptimizing previously calculated dose distributions with new penalty distributions.

Our penalty adaptation is based on an approach that scales the original penalties based on the objective function value, and therefore the dose of the respective voxel. We found that the adaptation of penalties within the healthy tissue by this method decreases the target coverage (and thus conformity) systematically since it also increases the local penalty near the target volumes. ΦWA, however, recognizes these areas as necessary to maintain target coverage and suppresses the originally calculated penalty scale $\phi_{i}^{O}$ for that voxel. The suppression strength is based on an approximation of the beam penumbra and on geometrical considerations represented by the allowed dose region Δδ. Within a parameter study we found that Δδ approximately equal to 12 mm yields good target coverage preservation for photon beams, which can be related to the bixel size of $5\;{\text{mm}}^{2}$ plus the finite number of beams and beamlets, limiting the geometrical freedom to sculpt dose to the target. Also the effect on OAR dose was found to be lower in general for the physically constrained strategy ${\phi_{i}}^{S}$ compared to the naive approach $\varphi^{O}$. This can be attributed to the suppressing nature of $\phi^{S}$ over $\varphi^{O}$.

We want to point out that our physical and geometrical considerations are only one part of the full picture. More physical constraints and additional *a priori* knowledge could be considered; for example, necessary entrance dose due to the photon depth‐dose curve could be integrated.

We applied ΦWA to generically optimized prostate and head and neck cases to evaluate the feasibility of the adaptation for class solution planning. For both treatment sites, we achieved preservation of the target coverage and elimination of undesired hot‐spots within the normal tissue. We did not alter the parameter set of α, $d_{T}$, and Δδ, suggesting a good transferability of the adaptation strategy within treatment sites with dose prescriptions of similar levels. We could, however, observe that the effect on coverage becomes larger with increasing plan complexity, as CO can drop more than one or two percentage points for few individual targets of the head and neck cases. Also the effects on OAR dose are not significantly smaller for $\phi^{S}$ over $\varphi^{O}$ (compare Fig. 8(d)). Still, we want to point out that, generally, the lower dose volumes increase, while the exposure to higher doses is slightly reduced.

Although we did not consider sequencing of the fluence maps into discrete multicollimator leaf segments in our study, the gained fluence maps showed qualitatively increased smoothness, which would facilitate the approximation of the fluence with a discrete number of apertures.

The results after repeated ΦWA adaptation become stable after only one or two adaptations and reoptimizations, with already the first reoptimization giving a practicable result. This leads to total run‐times of below 10 s for the prostate patients. Given the larger base data of the head and neck patients (compare Appendix A, Table A.1.) the run‐time increases, but stays below 30 s. From a clinical perspective, this high degree of automatization, combined with short runtimes, helps to reduce the workload per patient and objectifies planning.

In our study, we restricted the adaptation strategy solely on the body contour, excluding OARs and target volumes, to demonstrate the minimization of negative effects primarily on the targets by our physical adaptation. Developing physical and geometrical constraints for other VOI types is also possible. This is especially important for adapting plans generated with class solution constraint sets, since these are not optimal themselves, which can be seen in Appendix B, Tables B.3. and B.4. While our work aimed at efficient and fast reshaping of dose subject to elimination of undesired local artifacts while minimizing the effect on significant quality indicators, purposeful increase of coverage and simultaneous decrease of OAR exposure is of interest for enabling class solutions for the clinic. On the other hand, this could also be achieved by optimizing the class solution constraint sets on VOI‐basis beforehand.

Also, our adaptation strategy can be transferred to the optimization of other objectives, for example in direct aperture optimization or the optimization of dose‐volume measures in OARs or targets, if they are implemented as penalized soft‐constraints. Since dose‐volume measures are often implemented as penalized dose deviations as well, which are connected to a volume criterion via step functions,^(29)^ applying a comparable adaptation routine to the respective penalties in the affected voxels could be the next step in exploiting this method to achieve a similar effect on suppressing high‐dose regions in OARs or targets.

The fast and three‐dimensional GUI of DynaPlan enables the arbitrary manipulation of the penalty scale cube. This offers an extension of ΦWA with an intuitive interactive penalty adaptation method. For example, three‐dimensional brushes could be used to “paint” the respective adapted penalty scale distribution. First usage with Gaussian‐like brushes showed, however, that the free manipulation of the dose cube requires a lot more experience and intuition than working with globally calculated penalty distributions. The interactive method can, however, be used to fine‐tune penalty distributions fast and intuitively which were originally calculated with ΦWA .

## V. CONCLUSIONS

Voxel‐based penalty adaptation allows for exploration of a larger solution set than planning with VOI‐based objectives. The vast amount of possibilities to design a voxel‐based penalty distribution requires an automated generation. We presented an algorithm to constrain the penalty adaptation to the *a priori* known physical and geometrical limits of photon irradiation. Our algorithm effectively limits the negative effects of voxel‐based penalty adaptation regarding target coverage while still eliminating undesired high‐dose regions. We demonstrated that our algorithm can be used to improve class solutions for individual patients. Combined with ultra‐fast fluence optimization, this is a promising planning concept for adaptive online radiation therapy.

## ACKNOWLEDGMENTS

This work was supported by the Cancer Research UK Programme Grant C33589/A19727.

## COPYRIGHT

This work is licensed under a Creative Commons Attribution 3.0 Unported License.

## APPENDICES

### Appendix A: Planning Study

**Table A.1:** Base data information for the run‐time analysis, including number of voxels $n_{V}$, number of bixels $n_{b}$, and the size of the dose influence data $D_{\text{ij}}$. Other cases of the respective treatment site are of comparable size

*Case*	$n_{V}$	$n_{b}$	$D_{\text{ij}}$ *(MB)*
p 4	$4.5\times 10^{6}$	$1.4\times 10^{3}$	168
hn 1	$10.3\times 10^{6}$	$6.3\times 10^{3}$	457

### Appendix B: Class Solution Constraint Sets

**Table B.1:** Class solution constraint set for prostate patients

*VOI*	$D^{\text{max}}$ *(Gy)*	$p^{\text{max}}$	$D^{\text{min}}$ *(Gy)*	$p^{\text{min}}$
CTV	78	2500	78	2500
PTV	70	1500	70	4000
bladder	15	300	‐	‐
rectum	15	200	‐	‐
body contour	25	8000	‐	‐

**Table B.2:** Class solution constraint set for head and neck cases. The values for the chiasm and the cochlea nerve are listed below the dashed line since they were only delineated for one case and their radiation exposure could be neglected

*VOI*	$D^{\text{max}}$ *(Gy)*	$p^{\text{max}}$	$D^{\text{min}}$ *(Gy)*	$p^{\text{min}}$
PTV (70 Gy)	70	6000	70	10000
PTV (46 Gy)	46	4000	46	6000
spinal cord	10	100	‐	‐
brain	25	500	‐	‐
parotid gl.	10	500	‐	‐
subm. gl.	20	50	‐	‐
body contour	25	4000	‐	‐
opt. nerve	30	500	‐	‐
chiasm	30	500	‐	‐
coch. nerve	20	300	‐	‐

**Table B.3:** CO for all targets and mean and maximum doses for selected OARs of the class‐solution planned head and neck cases after one penalty adaptation run. For every case and QI, the top value represents the plan after the initial optimization. The second line indicates the values after reoptimization with $\phi^{S}$. The values in parentheses show results of reoptimization with $\varphi^{O}$

*VOI QI*	*70 Gy 1 CO (%)*	*70 Gy 2 CO (%)*	*70 G y3 CO (%)*	*46 GyL CO (%)*	*46 GyR CO (%)*	$D_{\text{mean}}$	*S. Cord* $D_{\text{max}}$	*Par. L* $D_{\text{mean}}$	*Par. R* $D_{\text{mean}}$	*Sub. L* $D_{\text{mean}}$	*Sub. R* $D_{\text{mean}}$	*Brain* $D_{\text{mean}}$	$D_{\text{max}}$
hn 1	91.0	89.7	‐	93.2	88.7	12.1	32.7	28.7^a^	15.0	66.9^a^	27.4	‐	‐
	89.2	89.0	‐	93.1	87.0	12.7	29.5	29.3^a^	15.6	66.9^a^	27.5	‐	‐
	(84.3)	(87.5)	‐	(91.7)	(84.0)	(12.7)	(30.3)	(29.3)^a^	(15.6)	(66.9)^a^	(26.8)	‐	‐
hn 2	88.7	92.1	‐	93.8	96.2	13.0	49.1	14.5	42.4^a^	32.7	68.2^a^	18.7	56.4
	87.3	91.6	‐	93.5	96.0	13.9	47.3	15.3	42.6^a^	32.4	68.2^a^	18.8	54.9
	(82.9)	(89.4)	‐	(92.1)	(94.6)	(13.9)	(44.4)	(15.3)	(42.9)^a^	(32.6)	(68.3)^a^	(18.1)	(53.1)
hn 3	92.6	95.0	‐	93.6	93.1	16.8	50.8	9.0	11.3	27.9	‐	16.2	52.5
	91.3	95.4	‐	93.6	92.9	17.0	46.5	10.0	12.8	28.0	‐	16.2	51.7
	(84.0)	(94.4)	‐	(91.7)	(91.0)	(16.3)	(43.0)	(10.2)	(13.5)	(27.8)	‐	(15.2)	(47.9)
hn 4	85.8	‐	‐	88.2	90.0	15.4	32.5	10.8	19.1	43.4^a^	44.1^a^	‐	‐
	85.3	‐	‐	87.9	89.6	15.3	32.1	10.9	19.1	43.5^a^	44.2^a^	‐	‐
	(79.8)	‐	‐	(83.9)	(87.2)	(15.6)	(31.8)	(10.8)	(19.0)	(43.4)^a^	(44.1)^a^	‐	‐
hn 5	88.5	95.5	98.6	96.7	89.3	19.6	47.7	17.9	12.5	69.3^a^	30.6	18.3	67.1
	88.4	94.8	98.5	96.9	88.6	19.2	44.4	19.1	13.3	68.7^a^	30.5	18.2	67.1
	(86.0)	(92.7)	(97.8)	(96.3)	(87.2)	(19.4)	(42.4)	(19.2)	(13.4)	(68.7)^a^	(31.1)	(17.6)	(63.5)

a ^a^ An OAR that could not be spared due to the full or large target overlap.

S.cord=spinal cord; par. 1/r=left/right parotid; sub. 1/r=left/right submandibular gland.

**Table B.4:** CO and CI for targets and dose‐volume indicators for the OARs of the prostate cases planned with the class solution constraint set after one reoptimization. For every case and QI, the top value represents the plan after the initial optimization. The second line indicates the values after reoptimization with $\phi^{S}$. The values in parentheses show results of reoptimization with $\varphi^{O}$

*VOI*	*PTV*		*CTV*		*Bladder*				*Rectum*				
*QI*	*CI*	*CO*	*CI*	*CO*	$V_{65\text{Gy}}$	$V_{70\text{Gy}}$	$V_{75\text{Gy}}$	$V_{80\text{Gy}}$	$V_{50\text{Gy}}$	$V_{60\text{Gy}}$	$V_{65\text{Gy}}$	$V_{70\text{Gy}}$	$V_{75\text{Gy}}$
p 1	93.5	94.2	91.1	97.5	5.2	3.6	1.2	0.0	14.0	10.1	8.4	6.0	3.0
	93.2	93.6	91.2	97.1	5.1	3.5	1.2	0.0	14.5	10.3	8.3	5.9	2.6
	(87.9)	(87.9)	(89.9)	(97.2)	(5.0)	(3.4)	(1.2)	(0.0)	(14.8)	(10.1)	(8.0)	(5.7)	(2.6)
p 2	92.5	93.7	90.4	95.8	9.6	7.3	4.5	0.0	26.0	20.3	17.0	12.6	5.0
	92.4	92.9	90.5	95.4	9.5	7.2	4.3	0.1	26.2	20.3	16.7	12.3	4.1
	(86.9)	(86.9)	(89.3)	(93.8)	(9.2)	(7.0)	(3.9)	(0.5)	(26.4)	(19.7)	(15.8)	(11.6)	(4.3)
p 3	94.3	95.1	91.1	96.4	3.0	2.3	1.3	0.0	6.5	4.3	3.5	2.4	0.9
	94.3	95.1	91.0	96.4	3.0	2.3	1.3	0.0	6.5	4.3	3.5	2.4	1.0
	(86.9)	(86.9)	(91.6)	(95.6)	(2.8)	(2.1)	(1.2)	(0.0)	(7.1)	(4.2)	(3.1)	(2.1)	(0.9)
p 4	94.0	96.6	90.2	96.9	5.1	3.7	1.9	0.0	4.6	3.1	2.8	1.5	0.5
	94.5	95.9	90.1	96.2	5.1	3.6	1.8	0.0	4.7	3.1	2.8	1.5	0.5
	(88.5)	(88.5)	(89.3)	(96.9)	(4.8)	(3.6)	(1.8)	(0.0)	(4.9)	(3.2)	(2.5)	(1.5)	(0.5)
p 5	85.6	86.4	66.6	99.2	20.7	13.8	3.1	0.0	24.0	18.3	14.7	9.9	1.2
	84.6	84.8	67.2	99.4	19.3	12.8	2.9	0.0	24.0	18.1	14.5	9.5	1.2
	(76.6)	(76.6)	(66.1)	(99.1)	(18.6)	(12.7)	(3.0)	(0.0)	(23.6)	(17.5)	(13.8)	(9.2)	(1.5)
p 6	93.9	95.4	85.4	99.6	8.2	6.3	3.8	0.1	19.0	14.9	12.7	9.8	4.2
	94.0	95.1	85.9	99.5	8.2	6.3	3.9	0.1	19.2	14.9	12.5	9.6	3.9
	(89.2)	(89.3)	(88.2)	(99.5)	(8.0)	(6.2)	(3.8)	(0.3)	(19.0)	(14.5)	(12.2)	(9.3)	(4.4)
p 7	94.0	94.8	91.5	95.9	4.0	2.9	1.4	0.0	15.0	10.8	8.6	5.9	2.4
	93.7	94.2	91.1	95.0	4.0	2.8	1.3	0.0	15.5	11.0	8.6	5.8	2.3
	(87.8)	(87.9)	(90.4)	(93.5)	(3.9)	(2.7)	(1.3)	(0.0)	(15.4)	(10.5)	(8.1)	(5.5)	(2.3)
p 8	87.9	89.1	81.1	95.0	20.1	12.9	1.7	0.0	16.8	12.9	10.2	5.6	0.2
	87.0	87.4	81.1	94.4	19.3	12.1	1.4	0.0	16.9	12.9	10.1	5.4	0.1
	(79.6)	(79.6)	(78.8)	(95.0)	(18.8)	(11.9)	(1.7)	(0.0)	(16.5)	(12.1)	(9.5)	(5.8)	(0.3)
p 9	92.4	93.9	90.0	96.5	5.3	4.0	2.6	0.0	19.6	15.8	12.7	10.8	6.5
	92.1	93.1	90.3	96.2	5.2	4.0	2.2	0.0	19.7	15.7	12.5	10.6	6.3
	(85.2)	(85.2)	(89.1)	(94.5)	(5.2)	(3.7)	(2.0)	(0.0)	(19.6)	(14.8)	(11.9)	(10.3)	(6.6)
p 10	92.8	94.2	90.0	95.5	8.4	6.5	3.7	0.1	16.0	12.0	9.9	7.2	3.7
	92.8	93.6	89.3	94.1	8.4	6.1	3.3	0.1	16.7	12.2	9.8	7.1	3.4
	(87.6)	(87.7)	(89.0)	(93.6)	(8.4)	(6.1)	(3.3)	(0.1)	(16.4)	(12.1)	(9.7)	(6.8)	(3.5)
p 11	90.6	93.1	79.2	95.5	3.0	1.9	0.4	0.0	10.8	7.0	4.8	2.2	0.4
	90.9	92.5	80.1	95.4	3.0	1.8	0.5	0.0	11.4	7.2	4.8	2.1	0.4
	(85.2)	(85.3)	(79.0)	(95.6)	(2.9)	(1.8)	(0.4)	(0.0)	(11.5)	(7.0)	(4.5)	(2.0)	(0.4)
p 12	93.1	94.6	88.9	96.2	4.5	3.3	1.8	0.1	19.1	14.5	12.3	8.8	3.9
	93.2	94.1	89.2	96.3	4.5	3.3	1.7	0.1	19.2	14.6	12.1	8.6	3.6
	(87.4)	(87.6)	(88.8)	(95.9)	(4.5)	(3.3)	(1.8)	(0.1)	(19.3)	(14.5)	(11.7)	(8.2)	(3.5)
p 13	93.6	95.3	91.5	95.4	3.9	3.0	1.8	0.0	20.3	14.8	11.7	8.4	3.6
	93.7	94.5	91.1	94.6	3.9	3.0	1.8	0.0	21.1	14.9	11.4	7.9	3.3
	(88.4)	(88.5)	(90.6)	(93.8)	(3.8)	(2.9)	(1.7)	(0.1)	(21.3)	(14.6)	(10.9)	(7.3)	(3.3)
p 14	93.1	93.9	90.4	95.6	8.9	7.1	4.7	0.5	21.7	16.5	13.7	10.1	4.5
	92.8	93.3	90.4	95.0	8.9	6.9	4.5	0.3	22.1	16.5	13.6	9.9	4.2
	(87.8)	(87.9)	(89.9)	(93.5)	(8.7)	(6.8)	(4.5)	(0.5)	(22.3)	(16.5)	(13.4)	(9.7)	(4.1)
p 15	93.1	94.5	90.9	95.7	7.2	5.6	3.6	0.1	18.9	14.6	12.1	8.9	3.5
	93.0	93.8	90.6	94.7	7.1	5.6	3.6	0.1	19.1	14.5	12.0	8.7	3.1
	(87.0)	(87.1)	(89.0)	(92.8)	(6.9)	(5.4)	(3.5)	(0.2)	(18.7)	(14.2)	(11.5)	(8.2)	(3.2)
p 16	92.8	94.3	90.5	96.8	98.3	90.7	75.7	1.2	23.8	18.5	15.7	11.9	5.9
	93.1	93.8	91.0	96.5	98.3	90.3	75.5	1.1	24.3	18.5	15.5	11.6	5.7
	(87.6)	(87.7)	(90.8)	(96.1)	(94.2)	(86.4)	(76.2)	(1.9)	(24.6)	(18.3)	(15.1)	(11.4)	(5.7)
